# Supplementary material for: Krüppel-like Factor (KLF) family members control expression of genes required for serous cavity and alveolar macrophage identities
Source: bioRxiv. 2024 Mar 3:2024.02.28.582578. Preprint. [Version 1] doi: 10.1101/2024.02.28.582578 (PMC10925242; doi:10.1101/2024.02.28.582578)
Supplement: Supplement 1 [file media-1.pdf]

Supplemental Figure 1- Pestal, et al

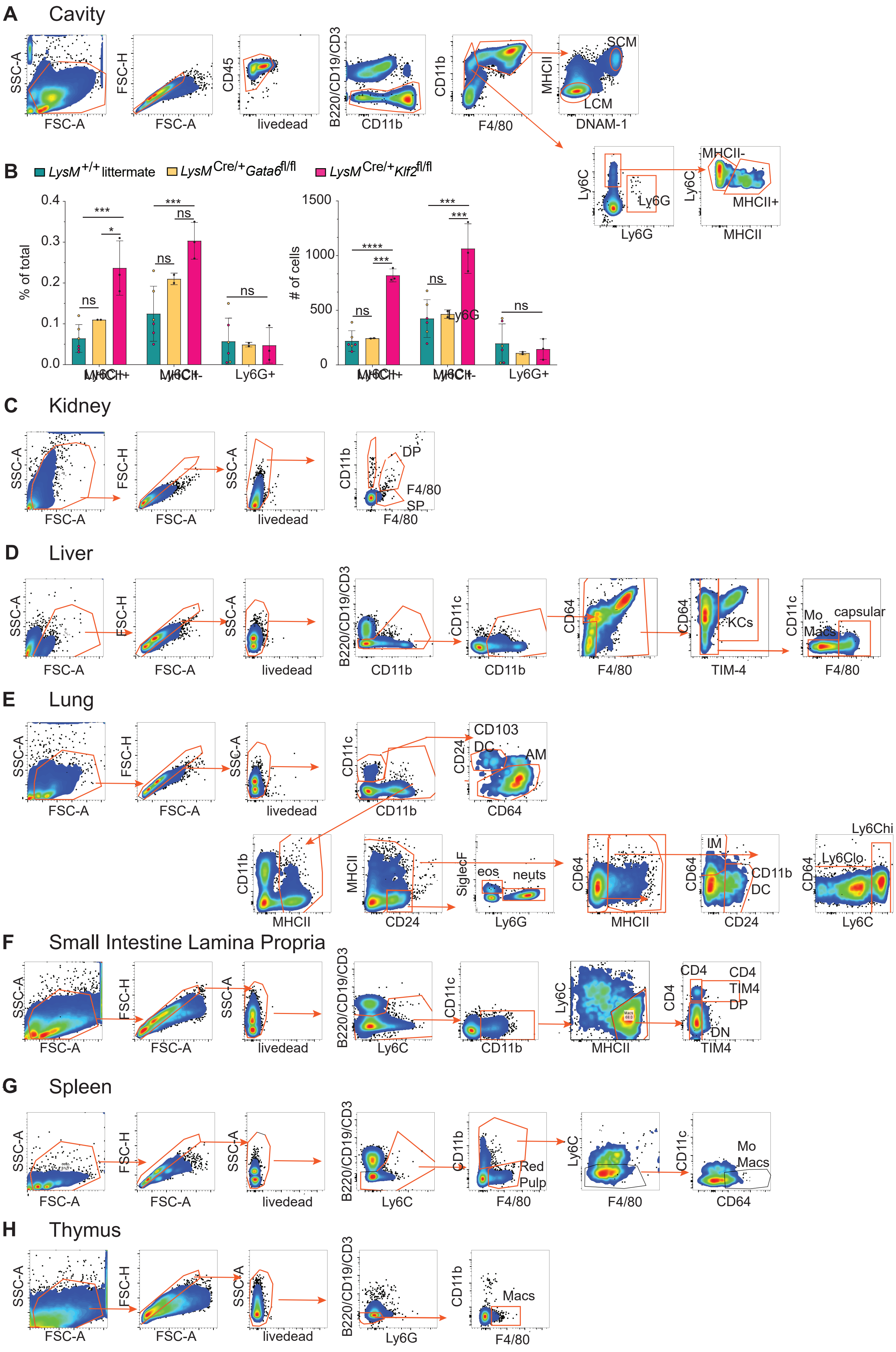

Fig S1

A: Flow cytometry gating strategy for cavities. **B:** Bar graphs depict the percent of total and total number of monocyte (CD11b$^+$F4/80$^-$MHCII$^+$ or MHCII$^-$) and neutrophils (CD11b$^+$F4/80$^-$, Ly6G$^+$), as measured by flow cytometry. Significance determined by ordinary 2-way ANOVA with multiple comparisons and Šidák's correction. Asterisks denote: ****<0.0001, *** 0.0006, ** 0.0021 , * 0.033). Flow cytometry gating for **C:** kidney, **D:** liver, **E:** lung, **F:** small intestine lamina propria, **G:** spleen, and **H:** thymus.

Supplemental Figure 2- Pestal, et al.

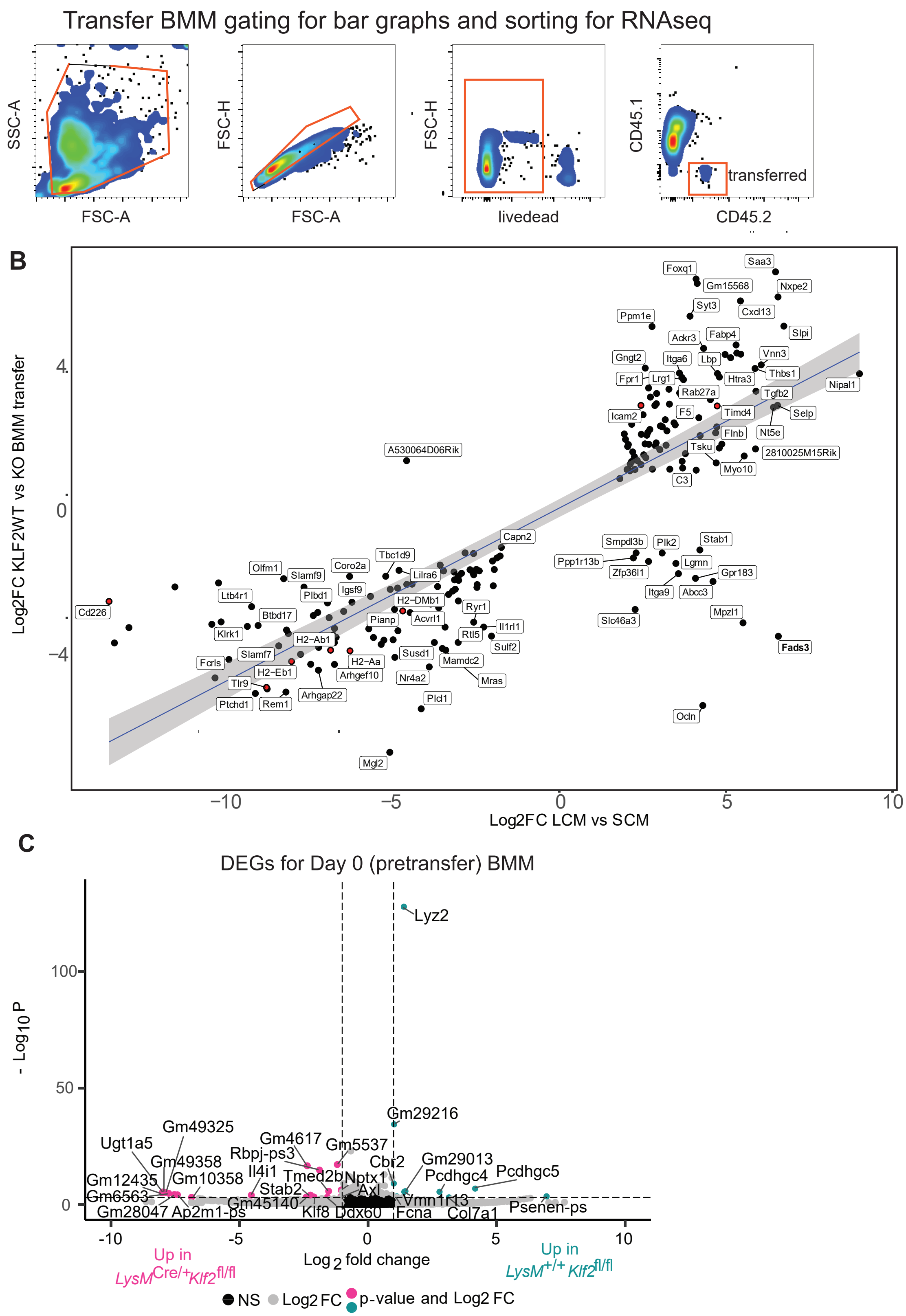

Fig S2

A: FACS gating for transferred bone marrow macrophages. **B:** Scatter plot of RNA Seq comparing differentially expressed genes between small and large cavity macrophages and transferred *LysM*$^{+/+}$*Klf2*$^{fl/fl}$ BMMs and *LysM*$^{Cre/+}$ BMMs with subset of genes labeled. **C:** Volcano plot of differentially expressed gene comparing pretransfer (day 0) *LysM*$^{+/+}$*Klf2*$^{fl/fl}$ to *LysM*$^{Cre/+}$*Klf2*$^{fl/fl}$ in vitro BMMs. Genes with a log2 Fold Change greater than |1| are colored grey. Genes with a p-value of -log10 (p value) greater than 4 are above the horizontal black line. Genes meeting both criteria are colored: teal, enriched in *LysM*$^{+/+}$*Klf2*$^{fl/fl}$; or pink, enriched in *LysM*$^{Cre/+}$*Klf2*$^{fl/fl}$.

Supplemental Figure 3- Pestal, et al

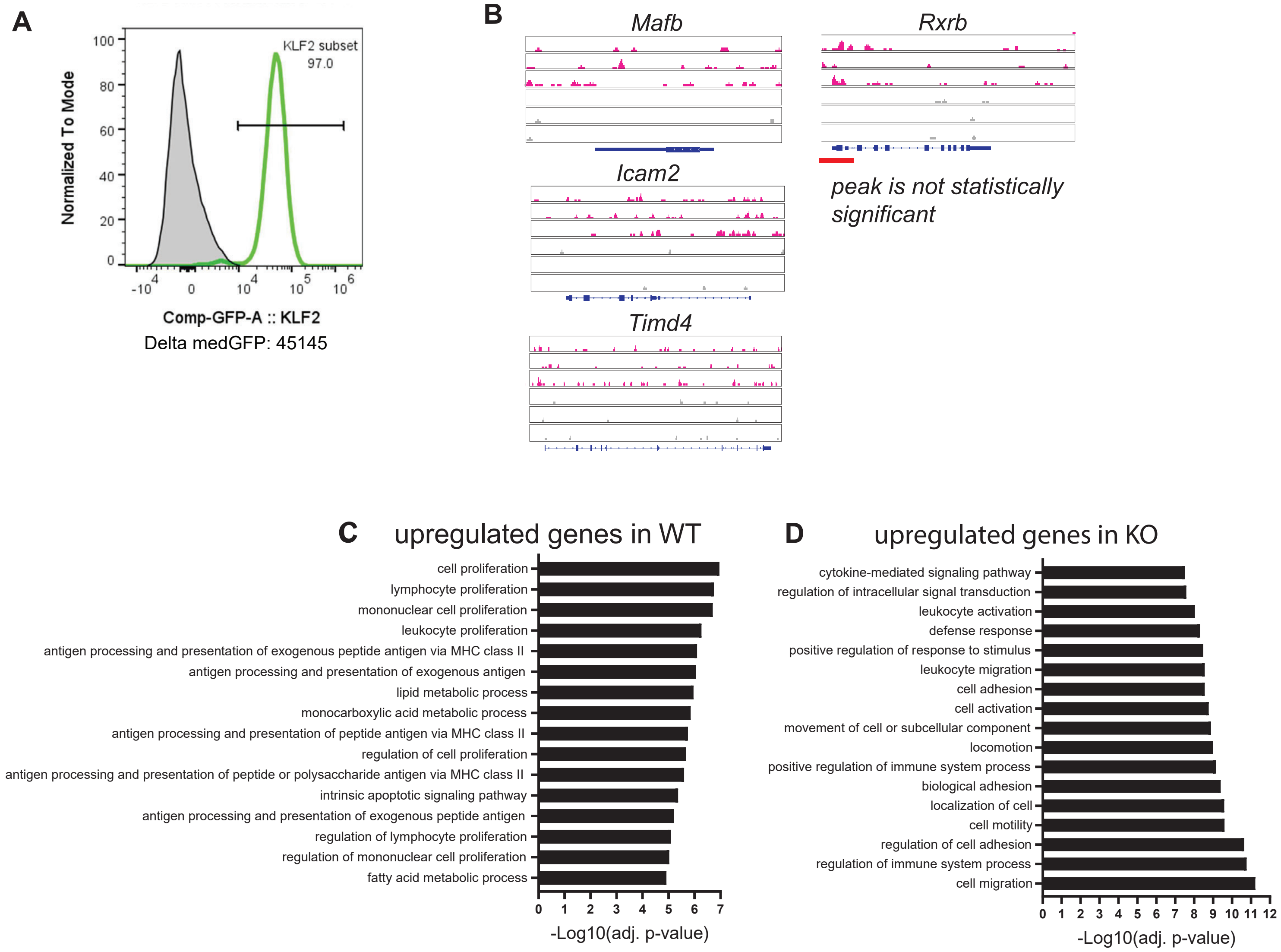

Fig S3

A: Representative flow cytometry of large cavity macrophages gated like SFig 1A (live, single, not B&T cells, F4/80^{+}CD11b^{+}DNAM1^{-}MHCII^{-}ICAM2^{+}) from GFP-KLF2 fusion positive mouse showing GFP expression. **B:** Genome browser tracks of anti-GPFKLF2 (pink) or IgG control (grey) CUT&RUN peaks from Large Cavity Macrophages in genes not directly regulated by KLF2 based on no peaks in the top 1% found by SEACR occur in two or more biological replicates. **C:** Top 25 GO term analysis results of genes upregulated in *LysM*$^{+/+}$*Klf4*$^{fl/fl}$ alveolar macrophages. **D:** Top 25 GO term analysis results of genes upregulated in *LysM*$^{Cre/+}$*Klf4*$^{fl/fl}$ alveolar macrophages

Supplemental Table 1- Pestal, et al

Marker	Clone(s)	Supplier:Fluorophore				
CD8	53-6.7	BD: BUV395				
I-A/I-E	M5/114.15.2	BD: BUV496	BioLegend: BV650			
CD80	16-10A1	BD: BUV563				
CD172a	P84	BD: BUV615				
CD102	3C4(mIC2/4)	BD: BUV661	BioLegend: AF647			
CD209b	22D1	BD: BUV737				
CD11b	M1/70	BD: BUV805	BioLegend: BV785			
Mer/MerTK	108928	BD: BV480				
H2 Class 1	M1/42	BD: BV510				
CD24	M1/69	BD: BV650	Thermo: SuperBright 436			
CD317	927	Thermo: BV786				
Siglec-H	440c	BD: BB700				
CD192	475301	BD: RB780				
TIM-4	21H12; RMT4-54	BD: R718	BioLegend: PE-Cy7	BioLegend: AF647		
CD19	6D5	Bio-Rad: StarBright SBV440	BioLegend: PE-Cy5			
F4/80	CI:A3-1; BM8	Bio-Rad: StarBright SBB580	BioLegend: PE	BioLegend: PE/Dazzle594		
CD3	KT3; 145-2C11	Bio-Rad: StarBright SBB615	BD: PE-Cy5			
NK1.1	PK136	Bio-Rad: StarBright SBB675				
Ly-6C	ER-MP20; HK1.4	Bio-Rad: StarBright SBB765	Thermo:APC-eF780	BioLegend: AF700	BioLegend: PE	Thermo: e450
CD45RA/B220	RA3-6B2	Bio-Rad: StarBright SBB810	BioLegend: PE-Cy5			
CD163	S15049I	BioLegend: BV421				
Ly-6G	1A8	BioLegend: Pacific Blue	BioLegend: AF700			
CD4	GK1.5	BioLegend: Spark Violet 538	Thermo: PE			
CD45	30-F11	BioLegend: BV570				
XCR1	ZET	BioLegend: BV605				
CD226	TX42.1; 10E5	BioLegend: BV711	BioLegend: PE/Dazzle594			
CD169	3D6.112	BioLegend: PE/Dazzle 594				
CD274	10F.9G2	BioLegend: PE/Fire 640				
CD86	GL1	BioLegend: PE-Cy5				
CD206	C068C2	BioLegend: PE/Fire 700				
CD64	X54-5/7.1	BioLegend: PE-Cy7				
CD103	QA17A24	BioLegend: PE-Fire 810				
CD11c	N418	BioLegend: Spark NIR 685	BioLegend: BV711			
CD16/32	S17011E	BioLegend: APC-Fire 750				
CX3CR1	SA011F11	BioLegend: APC/Fire 810				
MARCO	579511	R&D: APC				
LYVE-1	223322	R&D: Alexa Fluor 647				
Viability		Thermo: LIVE DEAD Blue	Thermo: LIVE DEAD Aqua			
Siglec-F	1RNM44N; E50-2440	Thermo: PerCP-eFluor 710	BD: AF647			
CD45.2	104	BioLegend: FITC				
CD45.1	A20	Thermo: APC-eF780				
GATA6	D61E4	Cell Signaling Tech: PE				

Table S1

Antibodies used for flow cytometry. First row of fluorophores used for high-parameter spectral flow cytometry on a 5 laser Cytek Aurora. Other fluorophores were used with traditional flow cytometry on a BD LSR Fortessa.
